# Effect of Cooking Oil on the Fatty Acid Profile of Beef Sausage Fortified with Edible Deboned Meat Waste

**DOI:** 10.1155/2021/5592554

**Published:** 2021-08-26

**Authors:** Babatunde O. Alao, Andrew B. Falowo, Elizabeth Bosede Aladejana

**Affiliations:** ^1^Department of Livestock and Pasture Science, Faculty of Science and Agriculture, University of Fort Hare, Alice, South Africa; ^2^Department of Animal Science, Faculty of Agriculture, Adekunle Ajasin University, Akungba Akoko, Nigeria; ^3^Medicinal Plants and Economic Development (MPED), Research Center, Faculty of Science and Agriculture, University of Fort Hare, Alice, South Africa

## Abstract

Application of cooking oil during thermal processing can influence the nutritional qualities of meat products during consumption. This study determined the effect of frying with sunflower and olive oil on the fatty acid profile of sausage fortified with edible meat waste (EMW) as a fat replacer was evaluated. Fresh beef sausages were formulated in ratios of 30% lean meat (LM) and 70% EMW, 50% LM and 50% EMW, and 90% LM and 10% fat (control) and designated as T1, T2, and T3, respectively. The proximate analysis results revealed significant differences (*P* < 0.05) in fat, fat free dry matter (FFDM), and moisture contents across the treatment. Fresh beef sausage fortified with 70% EMW had the highest fat contents (25.7 ± 0.83%) while those fortified with 10% fat (T3) had the highest FFDM (55.85 ± 0.57%) and moisture content (69.15 ± 0.62) compared to other treatments. In addition, among individual saturated fatty acids, beef sausage fortified with 50% meat wastes (T1) revealed significantly higher palmitic acid (31.06 ± 0.13), stearic acid (22.52 ± 0.29), myristic acid (3.84 ± 0.05), and lauric acid (0.04 ± 0.05) and the lowest margaric (0.98 ± 0.02) contents as compared to treatments T2 and T3. Also, beef sausage containing 10% fat showed the lowest (*P* < 0.05) saturated fatty acid (SFA) and higher monounsaturated (MUFA), polyunsaturated fatty acid (PUFA), n-6, n-3, PUFA : SFA, PUFA/MUFA, n-6/n-3, and desaturase indexes (DI) compared to treatments T1 and T2. Frying with sunflower oil significantly increased PUFA, n-6, n-6/n-3, and desaturase indexes and lowered SFA, n-3, and PUFA/SFA compared to frying with olive oil. In relation to raw beef sausage, frying with oil substantially increased the amount of MUFA, PUFA, n-6, and PUFA/SFA but reduced SFA content across the treatments.

## 1. Introduction

Meat is one of the most important sources of nutrients (protein, fat, essential amino acids, unsaturated fatty acids, minerals, vitamins, and other bioactive compounds) widely consumed by humans in both developed and developing countries across the world. The preference for meat and meat product consumption could be largely attributed to its taste, flavour, juiciness, palatability, and ability to provide high biological values in human diet [1–4]. Over the years, several production and culinary processes have been employed to improve meat palatability, tenderness, nutritional content, microbial safety, and inhibition of hazardous compounds during consumption [3]. The culinary process is often carried out by either frying, grilling, boiling, roasting, or microwaving [3]. However, frying of meat has been a common practice and accepted for a relatively long time due to better sensational qualities of the end products. Frying is a cooking method that helps to render potential organism inactive and increases nutrient availability of a product [5]. The temperature used for frying is normally between 170 and 200°C [3, 6]. During the course of frying, the oil and heat are transferred into the microstructure of the meat samples, while some water and volatile elements are evaporated from meat products, leaving a small part or a portion in the fried meat products [3, 6, 7]. Frying of meat often produces many changes such as modification or denaturation of protein, crust formation, loss of moisture, formation of aromatic compounds, and variation in colour which is produced through the Maillard reaction [3].

Also, Estévez et al. [8] and Ramirez et al. [9] in their studies reported that frying with vegetable oil causes alteration in the fatty acid profile and cooking loss. The chemical changes that occur during recurrent frying and within the oil are due to the formation of nonvolatile compound which often makes the product unsuitable for consumption as a result of nutritional disintegration [7]. The heat effect causes the cell structures of the meat to be disrupted and exposes a series of polyunsaturated fatty acids to lipid oxidation, hydrolysis, polymerization, and decomposition [10, 11]. The lipid polymers generated from oxidized products as a result of recurrent frying contain a significant quantity of cholesterol and saturated fatty acids [10]. Other factors influencing transmission of heat when meat products are being fried consist of the shape of the product, oil temperature, pressure, and chemical properties of the oil and the product [11]. Furthermore, during heating (frying), the polyunsaturated fatty acid (PUFA), especially the n-3 long-chain PUFA, is to a large extent more affected by oxidative deterioration of lipid that may cause discolouration and unpalatable flavour [12, 13].

Research studies indicated that vegetable oil represents a well-known dietary source of energy. The utilization of vegetable oil in meat products is effective in lowering the amount of saturated fatty acid contents thereby giving a positive effect on human health [14, 15]. Studies established that olive oil has beneficial effects in relation to nutritional value, a high level of oleic acid and small amount of linoleic acid, while sunflower oil has high level of linoleic acid (approximately 70%) although highly susceptible to oxidative changes [15, 16]. Any quantity of oil used in frying meat can either increase or reduce the fatty acid profile, and this depends on the type of oil used. A minimal amount of the fatty acid profile found in meat after frying implies that the oil may have good health implication [7, 10]. Therefore, an examination of the quantity of dietary fat and the ratio of saturated, monounsaturated, and polyunsaturated fatty acid in a fried product is important [17] in meat industry.

Presently, the interest of many researchers is to produce meat products, such as meat sausage, that can promote human health conditions. Meat sausages are usually made from ground meat (pork, beef, mutton, poultry, or veal) along with salt, spices, and other flavouring ingredients. In addition, products such as pork back fat, camel hump fat, beef fat, edible animal by-products, and vegetable oils have been incorporated into meat sausages with specific amount to enrich their textural and sensory quality and nutritional content and reduce their production cost [18, 19]. Therefore, the aim of the present study was to evaluate the effect of cooking oils on the fatty acid profile of beef sausages fortified with edible meat waste.

## 2. Materials and Methods

### 2.1. Description of the Study Site

The study was conducted in a commercial abattoir in East London situated under Buffalo City Metropolitan Municipality and Meat Science Laboratory at the University of Fort Hare. The East London abattoir is located 120 km away from Alice where the University of Fort Hare is established at latitudes and longitudes of 32.97°S and 27.87°E, respectively. The East London abattoir is a high throughput equipped with sophisticated machines and equipment fit for a standard abattoir.

### 2.2. Sample Collection and Beef Sausage Production

The lean beef meat and edible meat waste (EMW) were collected separately from slaughtered cattle at the abattoir to produce a novel beef sausage. Samples were packed in low-density polyethylene plastic bags and kept in a cooler and transported to the laboratory within 180 minutes. The EMW refers to meat waste generated during the cutting and trimming of the meat at the abattoir, and this usually consists of tendons, connective tissues, and trimmed meat fat. In the production of sausage, the lean beef and edible meat waste samples were combined in ratios of 30 : 70 and 50 : 50 and designated as T1 and T2, respectively, while the control contained 90% lean meat and 10% fat as treatment 3 (T3). Samples from each treatment were first chopped into 0.00129 meter square, mixed with seasonings (spices (0.33%), salt (0.19%), and ice (0.33%)), and then minced through a 5 mm plate using a mincer (TC22 EL ELEG.PLUS, Italy). Sausage batter was then pumped and stuffed lightly into a 25 mm diameter sheep sausage casing (Freddy Hirsch Company, Cape Town, South Africa) with Tre Spade sausage filler tools to produce average weight of 125 g each.

Fresh sausage from each treatment was then fried at 160-180°C for 5-10 minutes using two types of cooking oils (olive or sunflower oil). Doneness was determined by inserting a probe thermometer (ThermoPro TP food thermometer) into a geometrical centre of the sausage to measure its internal temperature during frying. The samples were considered done when the digital thermometer gave an alarm and flashed green light. The samples were drained, cooled at room temperature, vacuumed packed, and stored at -80°C until fatty acid analysis was carried out. All sausage samples were analysed in triplicate per treatment and per cooking oil type for fatty acid analysis.

### 2.3. Determination of Fatty Acid Composition

Total lipid of the raw and cooked sausage samples was quantitatively extracted as described by Folch et al. [20], using chloroform and methanol in a ratio of 2 : 1. An antioxidant (butylated hydroxytoluene (BHT)) was added at a concentration of 0.001% to the chloroform : methanol mixture. The fat extracts were dried in a rotary evaporator under vacuum, and the extracts were dried overnight in a vacuum oven at 50°C, using phosphorus pentoxide as the moisture absorbent. Total extractable fat was determined gravimetrically from the extracted fat and expressed as percent fat (*w*/*w*) per 100 g tissue. Thereafter, the extracted fat muscle was stored in a polytop (glass vial, with push-in top) under a blanket of nitrogen and frozen at –20°C for analysis of fatty acids.

A lipid aliquot (±30 mg) of sausage batter lipid was converted to methyl esters by base-catalyzed transesterification, in order to avoid CLA isomerization, with sodium methoxide (0.5 M solution in anhydrous methanol) during 2 h at 30°C, as proposed by Park et al. [21], Kramer et al. [22], and Alfaia et al. [23]. Fatty acid methyl esters (FAMEs) from sausage batter lipid were quantified using a Varian 430 flame ionization GC, with a fused silica capillary column, Chrompack CP-SIL 88 (100 m length, 0.25 mm ID, and 0.2 *μ*m film thicknesses). Analysis was performed using an initial isothermic period (40°C for 2 minutes). Thereafter, temperature was increased at a rate of 4°C/minute to 230°C. Finally, an isothermic period of 230°C for 10 minutes was followed. FAME n-hexane (1 *μ*l) was injected into the column using a Varian CP 8400 Autosampler. The injection port and detector were both maintained at 250°C. Hydrogen, at 45 psi, functioned as the carrier gas, while nitrogen was employed as the makeup gas. Galaxie Chromatography Data System Software recorded the chromatograms.

Fatty acid methyl ester samples were identified by comparing the retention times of FAME peaks from samples with those of standards obtained from Supelco (Supelco 37 Component Fame Mix 47885-U, Sigma-Aldrich Aston Manor, Pretoria, South Africa). Conjugated linoleic acid (CLA) standards were obtained from Matreya Inc. (Pleasant Gap, Unites States). These standards included cis-9 and trans-11 and trans-10 and cis-12-18:2 isomers. Heneicosenoic acid (C21:0) was used as the internal standard to improve quantitative FAME estimation.

Fatty acids were expressed as the proportion of each individual fatty acid to the total of all the fatty acids present in the sample. Fatty acid data were used to calculate the following ratios of FAs: total SFAs, total MUFAs, total PUFAs, PUFA/SFA, ∆^9^ desaturase index (C18:1*c*9/C18:0), total omega-6, total omega-3, and the ratio of omega-6 to omega-3 (*n*-6)/(*n*-3) FAs. The atherogenicity index (AI) was calculated as AI = (C12 : 0 + 4 × C14 : 0 + C16 : 0)/(MUFA + PUFA) [24].

### 2.4. Statistical Analysis

The Statistical Analysis System (SAS version 9.1.3 of 2007) was used for data analysis. The PROC GLM procedure of SAS was used to consider the effect of the types of cooking oil (olive oil and sunflower oil) and meat type (T1, T2, and T3) on the fatty acid beef sausage. Significant differences between the least square means for types of cooking oil were measured using Fishers' least significance difference (LSD) method of SAS, with a significance level of *P* < 0.05.

## 3. Results and Discussion

Result of the proximate analysis of the selected nutrients in raw beef sausages fortified with edible meat wastes is shown in Table 1. The result revealed significant differences (*P* < 0.05) in intramuscular fat, fat free dry matter (FFDM), and moisture contents across the treatment. As expected, beef sausage containing 70% edible meat wastes (T1) had higher fat content (25.7 ± 0.83%) than other treatments. Beef sausage fortified with 10% fat (T3) had the lowest fat content but highest FFDM (55.85 ± 0.57%) and moisture content (69.15 ± 0.62) than other treatments. A similar trend was also observed in the fried beef sausage (Table 2), with treatment containing 70% edible meat wastes (T1) having the highest fat content and lowest moisture content while treatment T3 had the lowest fat content and highest moisture content. The results of the study revealed that the fat content of the beef sausages in this study fell within the standard fat content of sausages [25]. Furthermore, Lee et al. [25] stated that the fat content of sausages can be as high as 30%. Meanwhile, findings emerging from this study showed that beef sausages cooked with sunflower oil had significant (*P* < 0.05) higher fat content (18.36%) and lower FFDM (25.93%) and moisture (55.91%) contents compared to those cooked with olive oil (Table 2). This observed increase in fat content of sausage cooked with sunflower oil could be attributed to the inherent fat and fatty acid content of each of the oil. Kuo and Gardner [26] had reported earlier that each cooking oil is composed of different fractions of fatty acid contents.

Table 3 shows the fatty acid composition of the beef sausages fortified with edible meat waste. There were significant differences (*P* < 0.05) in individual fatty acid composition of raw beef sausage across the treatments. Among individual saturated fatty acids, beef sausage fortified with 50% meat wastes (T1) revealed significantly higher palmitic acid (31.06 ± 0.13), stearic acid (22.52 ± 0.29), myristic acid (3.84 ± 0.05), and lauric acid (0.04 ± 0.05) and the lowest margaric (0.98 ± 0.02) contents compared to treatments T2 and T3. On the other hand, the individual monounsaturated fatty acid showed that beef sausage fortified with 10% fat (T3) had higher oleic (39.25 ± 0.34), vaccenic (0.52 ± 0.02), and heptadecenoic (0.67 ± 0.02) content and the lowest myristoleic (0.45 ± 0.01) and palmitoleic (2.50 ± 0.05) content as compared to treatments T1 and T2. The individual polyunsaturated fatty acid content was significantly higher in beef sausage containing 10% meat wastes (T3) as compared to treatments T1 and T2. In general, it was observed that the values of total monounsaturated fatty acid, polyunsaturated fatty acid, n-6, n-3, PUFA : SFA, PUFA/MUFA, n-6/n-3, and desaturase indexes (DI) were higher in beef sausage fortified with 10% fat than in other treatments. Meanwhile, the beef sausage fortified with 50% edible meat wastes (T2) had higher saturated fatty content and atherogenicity index (AI) compared to treatments T1 and T3. Thus, the findings in this study were in agreement with the Food and Agriculture Organization of the United Nations and World Health Organization, where the values of the n-3 : n-6 ratio and PUFA : SFA for raw beef sausages fell within the recommended ratio of ≥1 and >0.04, respectively, as required in human diet [27–31]. However, there is a dearth of literature to support the findings from this study because there continues to be scanty research work that has focused on evaluating the fatty acid composition of beef sausages fortified with edible meat waste.

As shown in Table 4, there were variations in fatty acid composition between samples (*P* < 0.05). As expected, beef sausage containing 10% fat (T3) had the lowest total SFA and highest MUFA and PUFA compared to treatments T1 and T2. In comparison to the raw beef sausage, cooking of the meat samples by frying significantly decreased the percentage of individual SFA values while increasing the individual MUFA and PUFA values as well as n-6, n-3, PUFA : SFA, PUFA/MUFA, n-6/n-3, and atherogenicity (AI) and desaturase index values across the treatments. These results are similar to the findings of Cunningham et al. [32] who reported that beef sausage cooked by pan frying had lower SFA content and higher MUFA and PUFA, compared to raw sausages. Furthermore, the cooking of beef sausages with olive oil greatly increased the content of individual saturated and monounsaturated fatty acidy compared to cooking with sunflower oil (*P* < 0.05). On the other hand, cooking of beef sausage with sunflower oil revealed higher PUFA, n-6, n-3, PUFA : SFA, PUFA/MUFA, n-6/n-3, and atherogenicity (AI) and desaturase index values than cooking with olive oil. These differences could be attributed to the individual fatty acid content of the cooking oil. A report has shown that the type of vegetable oil used during cooking can significantly influence changes in the fatty acid composition of meat products [33]. Sunflower oil used in this study is known to contain 9.40% SFA, 28.30% MUFA, 62.40% PUFA, 0.2% n-3 PUFAs, and 62.4% n-6 PUFAs [33], while olive oil contains 19.4% SFA, 68.2% MUFA, 18.0% PUFA, 1.6% n-3 PUFAs, and 16.4% n-6 PUFAs [34]. Other study has also shown that meat samples that were fried with canola cooking oil had higher n-6, MUFA, and PUFA content as compared to meat that was cooked through boiling and baking [35]. Similarly, Asmaa and Tajul [36] also found that chicken sausage fried with palm oil had higher fatty acid content than other treatments. All the authors attributed the increase in fatty acid content to the amount of oil retention on meat samples after cooking. Likewise, a recent study has also shown that people eating diet cooked with soybean oil significantly had higher serum *α*-linolenic acid concentrations compared to those eating diet cooked with sunflower oil [37].

## 4. Conclusion

Despite the increase in the omega-3 : omega-6 fatty acid ratio in the sausage treatments after frying with the oils, the mean value of the omega-3 : omega-6 fatty acid ratio was greater than 1 : 5 and this was within the FAO/WHO recommended range. Furthermore, the significant reduction in saturated fatty acids after cooking showed that the sausage could have a positive influence on the human health when consumed. Therefore, it may be concluded that using sausage made with edible meat waste as a fat replacer may not have any negative effects on human if consumed.

## Figures and Tables

**Table 1:** Proximate composition of the raw beef sausage fortified with edible animal wastes.

Parameters	Sausage type			*P* value
	T1	T2	T3	
Fat (%)	25.7 ± 0.83^a^	24.34 ± 0.70^a^	8.43 ± 0.76^b^	0.01
Fat free dry matter (%)	19.50 ± 0.22^a^	19.92 ± 0.19^a^	22.42 ± 0.20^b^	0.01
Moisture (%)	54.83 ± 0.67^a^	55.85 ± 0.57^a^	69.15 ± 0.62^b^	0.01

T1: beef sausage containing 30% lean beef and 70% EMW; T2: beef sausage containing 50% lean beef and 50% EMW; T3: beef sausage containing 90% lean beef and 10% fat. Significant: *P* ≤ 0.05, not significant: *P* > 0.05. Data were presented as means ± standard error of samples.

**Table 2:** Effect of cooking oil on the proximate composition of the beef sausage fortified with edible meat wastes.

Parameters	Sausage type (S)			Cooking oil type (O)		SEM	*P* value		O × S
	T1	T2	T3	Olive	Sunflower		O	S	
Fat (%)	23.5^a^	19.76^b^	9.31^c^	16.68	18.36	0.31	0.01	0.01	0.11
Fat free dry matter (%)	24.06^c^	25.67^b^	28.67^a^	26.32	25.93	0.23	0.23	0.01	0.09
Moisture (%)	52.44^c^	54.57^b^	62.05^a^	56.99	55.91	0.28	0.01	0.01	0.44

T1: beef sausage containing 30% lean beef and 70% EMW; T2: beef sausage containing 50% lean beef and 50% EMW; T3: beef sausage containing 90% lean beef and 10% EDMW; SEM: standard error of mean. Significant: *P* ≤ 0.05, not significant: *P* > 0.05. Note: the listed values in the column of olive and sunflower oils in this table are the pooled values of the effect of the oil type on the sausages.

**Table 3:** Fatty acid composition of the raw beef sausage fortified with edible animal waste.

Parameter	Sausage type			*P* value
	T1	T2	T3	
Saturated fatty acid (SFA)				
Lauric	0.03 ± 0.01^a^	0.03 ± 0.01^a^	0.02 ± 0.01^b^	0.02
Myristic	3.74 ± 0.05^a^	3.84 ± 0.05^a^	2.68 ± 0.05^b^	0.01
Pentadecylic	0.41 ± 0.01^a^	0.39 ± 0.01^a^	0.39 ± 0.01^a^	0.30
Palmitic	30.5 ± 0.15^b^	31.06 ± 0.13^a^	27.02 ± 0.14^c^	0.01
Margaric	1.07 ± 0.02^b^	0.98 ± 0.02^c^	1.26 ± 0.02^a^	0.01
Stearic acid	22.0 ± 0.34^a^	22.52 ± 0.29^a^	20.24 ± 0.31^b^	0.01
Arachidic	0.15 ± 0.03^a^	0.14 ± 0.004^a^	0.12 ± 0.004^b^	0.01
Total SFA	58.09 ± 0.46^a^	59.10 ± 0.39^a^	51.90 ± 0.42^b^	0.02
Monounsaturated fatty acids (MUFA)				
Myristoleic	0.66 ± 0.02^a^	0.65 ± 0.01^a^	0.45 ± 0.01^b^	0.01
Palmitoleic	3.02 ± 0.05^a^	3.09 ± 0.04^a^	2.50 ± 0.05^b^	0.01
Heptadecenoic	0.64 ± 0.02^b^	0.56 ± 0.02^c^	0.67 ± 0.02^a^	0.001
Vaccenic	0.22 ± 0.02^a^	0.20 ± 0.02^a^	0.52 ± 0.02^b^	0.01
Oleic	35.26 ± 0.37^b^	34.07 ± 0.32^c^	39.25 ± 0.34^a^	0.01
Erucic	0.02 ± 0.01^a^	0.23 ± 0.01^a^	0.12 ± 0.01^b^	0.01
Total MUFA	39.99 ± 0.42^b^	38.78 ± 0.36^c^	43.98 ± 0.39^a^	0.01
Polyunsaturated fatty acids (PUFA)				
Conjugated linoleic acid (CLA)	0.24 ± 0.01^a^	0.24 ± 0.01^a^	0.32 ± 0.01^b^	0.01
Linoleic	1.27 ± 0.07^b^	1.47 ± 0.06^c^	2.97 ± 0.06^a^	0.01
Linolelaidic	0.04 ± 0.01^a^	0.05 ± 0.01^a^	0.00 ± 0.01^b^	0.02
*α*-Linolenic	0.32 ± 0.01^b^	0.29 ± 0.01^c^	0.38 ± 0.01^a^	0.01
Eicosenoic	0.32 ± 0.00^a^	0.29 ± 0.00^a^	0.38 ± 0.00^a^	0.84
Elaidic	0.12 ± 0.01^a^	0.13 ± 0.01^a^	0.42 ± 0.01^b^	0.01
Phytanic	0.06 ± 0.00^a^	0.06 ± 0.00^a^	0.04 ± 0.00^b^	0.01
Arachidonic	0.06 ± 0.25^a^	0.09 ± 0.02^a^	0.41 ± 0.02^b^	0.01
Nonadecanoic	0.07 ± 0.01^a^	0.08 ± 0.01^a^	0.15 ± 0.01^b^	0.01
Docosapentaenoic	0.00 ± 0.01^a^	0.00 ± 0.01^a^	0.07 ± 0.01^b^	0.01
Total PUFA	1.93 ± 0.09^a^	2.12 ± 0.08^a^	4.12 ± 0.09^b^	0.01
n-6	1.61 ± 0.09^c^	1.83 ± 0.07^b^	3.69 ± 0.08^a^	0.30
n-3	0.32 ± 0.01^a^	0.31 ± 0.01^a^	0.43 ± 0.01^b^	0.01
PUFA : SFA	0.04 ± 0.00^a^	0.04 ± 0.00^a^	0.08 ± 0.00^b^	0.01
PUFA/MUFA	0.05 ± 0.00^c^	0.05 ± 0.00^b^	0.10 ± 0.00^a^	0.01
n-6/n-3	5.06 ± 0.21^c^	6.22 ± 0.18^b^	8.43 ± 0.12^a^	0.01
Atherogenic index	0.82 ± 0.01^b^	0.86 ± 0.01^a^	0.62 ± 0.01^c^	0.01
Desaturase index	1.60 ± 0.04^a^	1.52 ± 0.04^a^	1.96 ± 0.04^b^	0.01

T1: beef sausage containing 30% lean beef and 70% EMW; T2: beef sausage containing 50% lean beef and 50% EMW; T3: beef sausage containing 90% lean beef and 10% EDMW. Significant: *P* ≤ 0.05, not significant: *P* > 0.05. Data were presented as means ± standard error of samples.

**Table 4:** Effect of the cooking oil type on fatty acid composition of the beef sausages fortified with edible meat waste.

Parameters	Sausage type (S)			Cooking oil type (O)		SEM	*P* value		
	T1	T2	T3	Olive	Sunflower		O	S	O × S
Saturated fatty acid (SFA)									
Lauric	0.01^a^	0.03^b^	0.00^c^	0.02	0.01	0.003	0.37	<0.01	0.599
Myristic	3.59^b^	3.74^a^	2.60^c^	3.37	3.27	0.031	0.01	<0.01	0.176
Pentadecylic	0.38^a^	0.38^a^	0.37^a^	0.38	0.38	0.006	0.52	0.65	0.537
Palmitic	30.03^b^	31.12^a^	26.48^c^	29.45	28.96	0.158	0.04	<0.01	0.993
Margaric	1.01^b^	0.94^c^	1.19^a^	1.05	1.05	0.011	0.80	<0.01	0.738
Stearic acid	20.42^b^	20.94^a^	18.90^c^	20.07	20.11	0.159	0.87	<0.01	0.562
Arachidic	0.12b	0.13^a^	0.11^c^	0.11	0.12	0.002	0.02	<0.01	0.542
Total SFA	55.67^b^	57.69^a^	49.16^c^	54.58	54.02	0.280	0.167	<0.01	0.931
Monounsaturated fatty acids (MUFA)									
Myristoleic	0.71^b^	0.66^b^	0.02^a^	0.63	0.59	0.015	0.08	<0.01	0.810
Palmitoleic	3.26^a^	3.25^a^	2.58^b^	3.09	2.96	0.044	0.04	<0.01	0.441
Heptadecenoic	0.67^b^	0.57^c^	0.68^a^	0.65	0.63	0.007	0.16	<0.01	0.144
Vaccenic	0.26^b^	0.20^c^	0.48^a^	0.32	0.31	0.010	0.88	<0.01	0.600
Oleic	36.99^b^	34.34^c^	39.35^a^	37.18	36.61	0.258	0.12	<0.01	0.514
Erucic	0.02^b^	0.05^a^	0.18^c^	0.08	0.09	0.005	0.22	<0.01	0.763
Total MUFA	42.08^b^	39.31^c^	44.16^a^	42.21	41.49	0.295	0.093	<0.01	0.445
Polyunsaturated fatty acids (PUFA)									
Conjugated linoleic acid (CLA)	0.23^a^	0.20^a^	0.30^b^	0.25	0.24	0.010	0.39	<0.01	0.596
Linoleic	1.58^b^	2.65^c^	4.58^a^	2.29	3.57	0.255	<0.01	<0.01	0.241
Linolelaidic	0.01^a^	0.01^a^	0.01^a^	0.01	0.01	0.006	0.60	0.88	0.591
*α*-Linolenic	0.33^b^	0.31^a^	0.39^c^	0.34	0.34	0.005	0.93	<0.01	0.747
Eicosenoic	0.06^a^	0.06^ab^	0.04^b^	0.05	0.05	0.005	0.74	0.05	0.596
Elaidic	0.13^a^	0.19^a^	0.39^b^	0.22	0.25	0.022	0.43	<0.01	0.441
Phytanic	0.06^a^	0.05^b^	0.04^c^	0.05	0.05	0.001	0.55	<0.01	0.542
Arachidonic	0.09^a^	0.13^a^	0.62^b^	0.28	0.28	0.019	0.80	<0.01	0.595
Nonadecanoic	0.06^a^	0.06^a^	0.13^b^	0.08	0.09	0.003	0.14	<0.01	0.514
Docosapentaenoic	0.00^a^	0.00^a^	0.11^b^	0.03	0.04	0.005	0.54	<0.01	0.691
Total PUFA	2.23^c^	3.29^b^	6.02^a^	3.21	4.49	0.251	0.0012	<0.01	0.248
n-6	1.90^c^	2.99^b^	5.52^a^	2.83	4.10	0.252	0.0012	<0.0001	0.245
n-3	0.33^a^	0.31^a^	0.51^c^	0.38	0.38	0.009	0.717	<0.0001	0.859
PUFA : SFA	0.04^a^	0.06^a^	0.12^b^	0.06	0.09	0.005	0.0019	<0.0001	0.260
PUFA/MUFA	0.05^c^	0.09^b^	0.14^a^	0.08	0.11	0.007	0.0014	<0.0001	0.180
n-6/n-3	5.74^ab^	9.94^a^	10.98^a^	7.16	10.62	0.714	0.002	<0.0001	0.156
Atherogenic index	0.76^b^	0.82^a^	0.58^c^	0.73	0.71	0.008	0.066	<0.0001	0.100
Desaturase index	1.81^b^	1.64^c^	2.08^a^	1.86	1.83	0.02	0.381	<0.0001	0.712

T1: beef sausage containing 30% lean beef and 70% EMW; T2: beef sausage containing 50% lean beef and 50% EMW; T3: beef sausage containing 90% lean beef and 10% EDMW; SEM: standard error of mean. Significant: *P* ≤ 0.05), not significant: *P* > 0.05. Note: the listed values in the column of olive and sunflower oils in this table are the pooled values of the effect of the oil type on the sausages.

## Data Availability

The data used to support the findings of this study are available from the corresponding author upon request.
